# Evaluation of Apixaban safety and effectiveness in morbidly obese patients with atrial fibrillation: a retrospective cohort study

**DOI:** 10.1186/s12959-022-00379-x

**Published:** 2022-05-02

**Authors:** Khalid Al Sulaiman, Hisham A. Badreldin, Ghazwa B. Korayem, Abeer A. Alenazi, Faisal Alsuwayyid, Abdulrahman Alrashidi, Mohammed Alhijris, Faisal Almutairi, Fahad Alharthi, Ramesh Vishwakarma, Omar Al Shaya, Abdulrahman Al Amri, Saqiba Tayyab, Abdulkareem M. Al Bekairy, Ohoud Aljuhani

**Affiliations:** 1 Pharmaceutical Care Department, King Abdulaziz Medical City, Riyadh, Saudi Arabia; 2 Saudi Critical Care Pharmacy Research (SCAPE) Platform, Riyadh, Saudi Arabia; 3 College of Pharmacy, King Saud bin Abdulaziz University for Health Sciences, Riyadh, Saudi Arabia; 4 King Abdullah International Medical Research Center, Riyadh, Saudi Arabia; 5 Department of Pharmacy Practice, College of Pharmacy, Princess Nourah bint Abdulrahman University, P.O.Box 84428, Riyadh 11671, Saudi Arabia; 6 Pharmaceutical Care Department, Prince Sultan Military Medical City, Riyadh, Saudi Arabia; 7 Statistics Department, European Organization for Research and Treatment of Cancer (EORTC) Headquarters, Brussels, Belgium; 8 Pharmaceutical Care Department, Care national hospital, Riyadh, Saudi Arabia; 9 Department of Pharmacy Practice, Faculty of Pharmacy, King Abdulaziz University, Jeddah, Saudi Arabia

**Keywords:** Apixaban, Morbidly obese, BMI ≥ 40, Atrial fibrillation, Thrombosis, Bleeding

## Abstract

**Background:**

The benefit of apixaban to reduce stroke risk in morbidly obese patients with nonvalvular atrial fibrillation (AF) is still undetermined. The International Society of Thrombosis and Hemostasis recommends avoiding the use of direct oral anticoagulants (DOAC)s in morbidly obese patients (body mass index > 40 or weight > 120 kg) because of limited clinical data. This exploratory study aims to evaluate the effectiveness and safety of using apixaban in morbidly obese (body mass index (BMI) ≥ 40) patients with AF.

**Methods:**

An exploratory retrospective cohort study was conducted at a single-center, including adult patients with non-valvular AF using apixaban between 01/01/2016 and 31/12/2019. Patients were excluded if they were known to have liver cirrhosis Child-Pugh C, mechanical valve, serum creatinine > 1.5 mg/dL, follow up < 3 months, or using apixaban with a dose of ≤5 or > 10 mg/day. Included patients were categorized into two groups based on their BMI (BMI<40 *Vs*. BMI ≥ 40). The primary outcome was all thrombotic events, while the secondary outcomes were major and minor bleeding after apixaban initiation. Propensity score (PS) matching was used (1:1 ratio) based on the patient’s age, gender, and HAS-BLED score.

**Results:**

A total of 722 patients were eligible; 254 patients were included after propensity score matching based on the selected criteria. The prevalence of all thrombotic events was similar between the two groups in the first year of apixaban initiation (OR (95%CI): 0.58 (0.13, 2.5), *p*-value = 0.46). In addition, the odds of developing major and minor bleeding were not statistically significant between the two groups (OR (95%CI): 0.39 (0.07, 2.03), *p*-value = 0.26 and OR (95%CI): 1.27 (0.56, 2.84), p-value = 0.40), respectively).

**Conclusion:**

This exploratory study showed similar effectiveness and safety of apixaban use in both morbid and non-morbid obese patients with non-valvular AF. However, a larger randomized controlled trial with a longer follow-up period needs to confirm our findings.

## Background

Recent guidelines recommend using direct oral anticoagulants (DOACs) as first-line oral agents over warfarin in patients with non-valvular Atrial fibrillation (AF) and elevated stroke risk [1, 2]. DOACs have proven efficacy in stroke and systemic embolism risk reduction while overcoming many warfarin drawbacks [3, 4]. DOACs advantages over warfarin include better pharmacokinetics, early-onset, fewer routine drug monitoring, and fewer interactions [5]. In addition, DOACs have a favorable safety profile in special populations such as patients with renal dysfunction, patients with history of stroke, or older adults [6]. However, because overweight and obese adults are often underrepresented in trials, the safety and effectiveness of DOACs in obese patients are unknown [6–9]. Therefore, a guidance issued by the International Society on Thrombosis and Hemostasis (ISTH) recommends against the use of DOAC in patients with a BMI > 40 kg/m^− 2^ or weight above 120 kg due to limited evidence [10].

Since that recommendation, several studies and post-hoc analyses have investigated the impact of BMI on the safety and effectiveness DOACs, including Dabigatran, Rivaroxaban, Apixaban, and Edoxaban [11–16]. A systemic review and meta-analysis including a total of 89,494 morbidly obese patients with non-valvular AF comparing DOACs to warfarin reported that DOAC was associated with significantly lower stroke or systemic embolism [odds ratio (OR): 0.71; 95% confidence interval (CI): 0.62–0.81; *P* < 0.0001; I^2^ = 0%] compared to warfarin [17]. In addition, DOACs had a significantly lower major bleeding compared to warfarin (OR: 0.60; 95% CI: 0.46–0.78; *P* < 0.0001; I^2^ = 86%) [17]. A sub-class analysis in that meta-analysis showed that apixaban and rivaroxaban were superior to warfarin in safety and efficacy [17]. Although the results of several meta-analyses support the use of rivaroxaban and apixaban in morbidly obese patients with non-valvular AF, additional randomized clinical trials comparing the efficacy and safety of DOACs to warfarin in this patient population are needed before a strong recommendation can be made [17, 18].

Apixaban and other DOACs have been proposed as an alternative to warfarin for non-valvular AF or flutter stroke prevention in morbidly obese patients [15, 19]. This recommendation mainly derives from post-hoc analyses, meta-analyses, retrospective, observational, or small non-randomized studies [11–18]. The efficacy and safety of apixaban in patients with extreme weights are still being investigated [20]. Thus, we conducted this exploratory study to evaluate the efficacy and safety of using apixaban in AF patients who are morbidly obese (BMI ≥ 40).

## Methods

### Study design

This was an exploratory retrospective cohort study retrieving adult patients’ data from the electronic health records of King Abdulaziz Medical City (Riyadh) between January 01st, 2016 and December 31st, 2019. We included adult patients aged 18 years or above diagnosed with non-valvular AF and who received apixaban for stroke prevention regardless of their CHA2DS2-VASc score. Patients were excluded if they are known to have liver cirrhosis Child C, mechanical valve, serum creatinine > 1.5 mg/dL, follow up < 3 months, or using apixaban with a dose of ≤5 or > 10 mg/day. Included patients were then categorized into two groups based on the body mass index (BMI) (BMI ≥ 40 vs. BMI < 40) at the time of apixaban initiation. Patients' data were then followed for at least one year after apixaban initiation. The Institutional review board at King Abdullah International Medical Research Center (KAIMRC) approved the study in November 2021 (Ref.# NRC21R/482/11). Informed consent from the study patients was waived due to the retrospective observational nature of the study.

### Study setting

This study was conducted in King Abdulaziz Medical City (Riyadh). It is a tertiary-care academic referral hospital with 1601 beds in Riyadh, Saudi Arabia. King Abdulaziz Medical City includes cardiac and liver centers, intensive care units (ICUs), dental, ambulatory care centers, long-term care/ rehabilitation, emergency care, trauma centers, surgical and medical wards, obstetrics and gynecology, pediatrics, operating rooms, and home health care program.This center provides all types of care to all national guard soldiers and their families, including primary health care and specialized tertiary care.

### Data collection

Each patients’ data were collected and retrieved from the hospital system (Best Care); data were collected in an excel sheet. We collected patients’ demographic data, comorbidities, vital signs and laboratory tests, CHA2DS2-VASc score, and HAS-BLED scores at the time of apixaban initiation. Moreover, laboratory tests including coagulation profile, liver, and renal profile, complete blood count (hemoglobin and platelets), concomitant antiplatelets use, and concomitant use of gastrointestinal (GI) prophylaxis were collected. Moreover, we collect the following variables: confirmed stroke occurrence (ischemic or hemorrhagic), pulmonary embolism (PE), deep vein thrombosis (DVT), upper or lower GI bleeding (confirmed by documentation or using upper endoscopy or colonoscopy), the occurrence of left ventricular thrombus (confirmed by echocardiogram) while on apixaban.

### Outcomes

This exploratory study aims to compare the effectiveness and safety of apixaban in patients with non-valvular AF who are morbidly obese (BMI ≥ 40) to non-morbidly obese patients (BMI < 40). The primary efficacy outcome was thrombotic events. Thrombotic events were identified using the International Classification of Diseases, Tenth Revision, Clinical Modification (ICD10-CM) code (i.e., stroke, pulmonary embolism, deep vein thrombosis), chart review documentation and/or radiology findings. In contrast, the secondary outcomes were major and minor bleeding after apixaban initiation. Major bleeding was defined according to the ISTH as clinically overt bleeding associated with a fall in hemoglobin by ≥20 g/L, transfusion of ≥2 U packed red blood cells (PRBCs) or whole blood, retroperitoneal or intracranial bleeding, or fatal bleeding [21]. In comparison, minor bleeding was defined according to the ISTH definition as any sign or symptom of bleeding that does not fit the criteria for the ISTH definition of major bleeding [21], but does meet at least one of the following criteria: requiring medical intervention by a healthcare professional, bleeding leading to hospitalization or increased level of care or prompting a face to face evaluation [22].

### Statistical analysis

We presented continuous variables as mean with standard deviation (SD), or median with lower and upper quartile (Q1, Q3) as appropriate and categorical variables as number (percentage). The normality assumptions were assessed for all numerical variables using a statistical test (i.e., Shapiro–Wilk test) and graphical representation (i.e., histograms and Q-Q plots).

Baseline characteristics and outcome variables were compared between the two study groups. For categorical variables, we used the Chi-square or Fisher’s exact test. We compared the normally distributed continuous variables using the student t-test and non-normally distributed variables with the Mann-Whitney U test. Multivariable regression analysis and negative binomial regression were used for the outcomes considered in this study as appropriate. Regression analysis was done by considering PS score as one of the covariates in the model. The odds ratios (OR) or estimates with the 95% confidence intervals (CI) were reported as appropriate. No imputation was made for missing data as the cohort of patients in our study was not derived from random selection. We considered a *P* value of < 0.05 statistically significant and used SAS version 9.4 for all statistical analyses.

Propensity score matching procedure (Proc PS match) (SAS, Cary, NC) was used to match patients with BMI ≥ 40 (active group) to patients with BMI<40 (control group) who received Apixaban therapy based on patient’s age, gender, and HAS-BLED score at the time of Apixaban initiation. A greedy nearest neighbor matching method was used in which one patient with BMI ≥ 40 (active) group matched with one patient with BMI<40 (control), which eventually produced the smallest within-pair difference among all available pairs with treated patients. Patients were matched only if the difference in the logits of the propensity scores for pairs of patients from the two groups was less than or equal to 0.5 times the pooled estimate of the standard deviation.

## Results

### Demographic and clinical characteristics

**Fig. 1:**
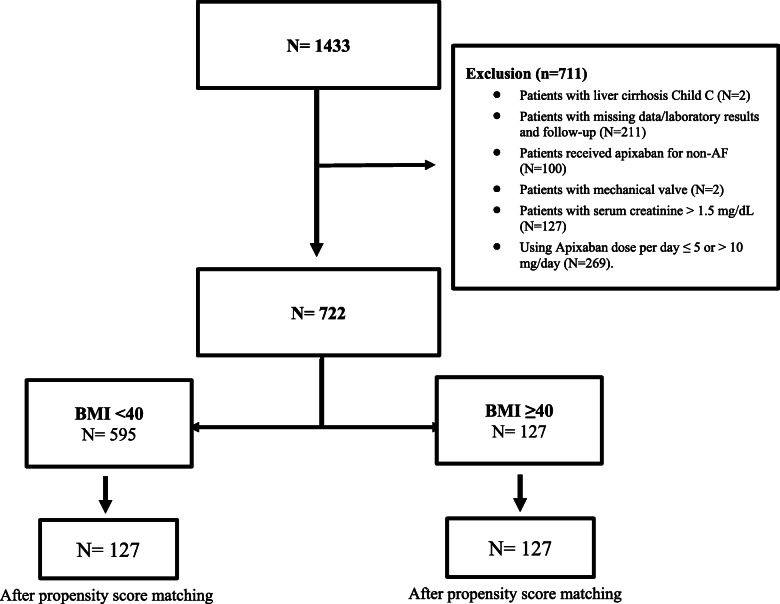
Flowchart of patients who received apixaban

A total of 1433 patients were screened after applying the selection criteria. However, only 722 out of them met the inclusion criteria and received apixaban for non-valvular AF. We matched 254 patients using propensity score (1:1) according to the selected criteria. Then patients were divided based on their BMI to < 40 kg/m^ 2^ and ≥ 40 kg/m^ 2^ as presented in the patient’s flow chart (Fig. 1). All patients received a fixed dose of apixaban (5 mg twice daily). Before PS matching, patients with BMI < 40 were older, male, had a higher eGFR, INR, aPTT, and stroke prevalence. However, after using PS matching, most of the baseline characteristics and comorbidities were balanced between the two groups. Except for higher hematocrit levels in patients with BMI ≥40 kg/m^ 2^ and longer aPTT level among patients with BMI < 40 kg/m^ 2^. The mean weight in patients with BMI < 40 after PS matching was 75.2 kg (±12.47) compared to 108.4 kg (±17.72) in the active group (Table 1).

**Table 1:** Baseline characteristics of adult patients receiving apixaban before and after propensity score matching

	Before propensity score (PS) matching				After propensity score (PS) matching			
	Overall (***N*** = 722)	BMI < 40 (***N*** = 595)	BMI ≥ 40 (***N*** = 127)	*P*-value	Overall (***N*** = 254)	BMI < 40 (***N*** = 127)	BMI ≥ 40 (***N*** = 127)	*P*-value
**Age (Years), Mean (SD)**	69.9 (11.39)	70.2 (11.56)	68.3 (10.46)	0.0212^	68.3 (10.31)	68.3 (10.20)	68.3 (10.46)	0.9806*
**Gender – Male, n (%)**	307 (42.5)	291 (48.9)	16 (12.6)	<.0001^^	32 (12.6)	16 (12.6)	16 (12.6)	0.9999^^
**Weight (kg), Mean (SD)**	83.4 (18.90)	78.1 (14.33)	108.4 (17.72)	<.0001*	91.8 (22.59)	75.2 (12.47)	108.4 (17.72)	<.0001*
**Body Mass Index (BMI), Median (Q1, Q3)**	32.1 (27.33, 37.86)	30.5 (26.63, 34.57)	43.9 (41.71, 47.74)	<.0001*	39.8 (32.45, 43.86)	32.5 (27.18, 35.41)	43.9 (41.71, 47.74)	<.0001*
**Alanine aminotransferase (ALT), Median (Q1, Q3)**	21.0 (14.00, 31.00)	21.0 (14.00, 31.00)	19.0 (13.00, 25.00)	0.0551^	19.5 (13.00, 28.00)	20.0 (13.00, 29.50)	19.0 (13.00, 25.00)	0.7210^
**Aspartate aminotransferase (AST), Median (Q1, Q3)**	22.0 (17.00, 29.00)	22.0 (17.00, 29.00)	21.0 (16.00, 28.00)	0.1182^	21.0 (16.00, 28.00)	21.5 (15.50, 27.00)	21.0 (16.00, 28.00)	0.9873^
**Estimated glomerular filtration rate (eGFR), Median (Q1,Q3)**	83.0 (69.00, 97.00)	83.0 (69.50, 98.00)	80.0 (63.00, 92.00)	0.0229^	81.0 (67.00, 94.00)	83.0 (71.00, 97.00)	80.0 (63.00, 92.00)	0.1693*
**Serum creatinine (mmol/l), Median (Q1,Q3)**	73.0 (64.00, 90.00)	73.5 (64.00, 90.00)	70.0 (63.00, 85.00)	0.1956^	69.0 (62.00, 82.00)	69.0 (61.00, 77.00)	70.0 (63.00, 85.00)	0.1698^
**Blood Urea Nitrogen (BUN) (mmol/l), Median (Q1,Q3)**	5.6 (4.30, 7.30)	5.6 (4.30, 7.20)	5.7 (4.10, 7.85)	0.8249^	5.3 (4.00, 7.30)	5.1 (4.00, 6.60)	5.7 (4.10, 7.85)	0.1059^
**Platelets count (10^9/L), Median (Q1, Q3)**	245.0 (203.00, 312.00)	244.5 (203.00, 309.50)	255.0 (203.00, 333.00)	0.2074^	261.0 (205.50, 335.00)	264.0 (209.00, 335.00)	255.0 (203.00, 333.00)	0.6115^
**Hematocrit (Hct), Median (Q1, Q3)**	0.4 (0.35, 0.44)	0.4 (0.35, 0.44)	0.4 (0.37, 0.44)	0.4713^	0.4 (0.35, 0.43)	0.4 (0.35, 0.42)	0.4 (0.37, 0.44)	0.0366^
**Total WBC (10^9/L), Median (Q1, Q3)**	7.8 (6.10, 10.00)	7.7 (6.10, 10.00)	8.1 (6.40, 9.94)	0.4107^	8.0 (6.17, 10.05)	7.9 (6.10, 10.10)	8.1 (6.40, 9.94)	0.6404^
**International normalized ratio (INR), Median (Q1,Q3)**	1.1 (1.04, 1.20)	1.1 (1.05, 1.22)	1.1 (1.02, 1.14)	0.0010^	1.1 (1.03, 1.15)	1.1 (1.04, 1.20)	1.1 (1.02, 1.14)	0.2394^
**Activated partial thromboplastin time (aPTT) (Seconds), Median (Q1, Q3)**	29.9 (27.20, 34.40)	30.1 (27.45, 34.75)	28.8 (26.40, 33.10)	0.0058^	29.4 (26.80, 33.90)	29.8 (27.40, 34.40)	28.8 (26.40, 33.10)	0.0453^
**Total bilirubin (μmol/L), Median (Q1, Q3)**	10.7 (8.10, 14.80)	10.7 (8.10, 15.00)	10.7 (7.80, 13.30)	0.3140^	9.8 (7.40, 12.90)	9.1 (7.10, 12.70)	10.7 (7.80, 13.30)	0.0805^
**Albumin (gm/L), Median (Q1, Q3)**	37.0 (33.00, 40.00)	37.0 (32.00, 40.00)	36.0 (33.00, 39.00)	0.6269^	37.0 (33.00, 39.00)	37.0 (33.00, 40.00)	36.0 (33.00, 39.00)	0.2868^
**Fibrinogen Level (gm/l), Median (Q1,Q3)**	3.9 (2.59, 5.16)	3.9 (2.29, 5.00)	5.5 (4.63, 6.18)	0.0494*	4.8 (1.64)	4.2 (1.96)	5.4 (1.02)	0.3234*
**D-dimer Level (mg/l), Median (Q1,Q3)**	1.1 (0.47, 2.92)	1.2 (0.50, 3.08)	1.0 (0.47, 2.74)	0.7596^	1.1 (0.46, 2.86)	1.1 (0.42, 3.70)	1.0 (0.47, 2.74)	0.9782^
**Blood sugar level (mmol/L), Median (Q1, Q3)**	7.8 (5.90, 11.40)	7.7 (5.90, 11.20)	8.8 (6.10, 11.80)	0.1628^	8.7 (6.00, 12.50)	8.4 (5.90, 13.20)	8.8 (6.10, 11.80)	0.7573^
**CHA2DS2-VASc Score, Median (Q1, Q3)**	4.0 (3.00, 5.00)	4.0 (3.00, 5.00)	4.0 (3.00, 5.00)	0.5234^	4.0 (3.00, 5.00)	4.0 (3.00, 5.00)	4.0 (3.00, 5.00)	0.8711^
**HAS-BLED Score, Median (Q1, Q3)**	2.0 (1.00, 3.00)	2.0 (1.00, 3.00)	2.0 (1.00, 3.00)	0.0993^	2.0 (1.00, 2.00)	2.0 (1.00, 2.00)	2.0 (1.00, 3.00)	0.5930^
**History of Major Bleeding, n (%)**	4 (0.6)	3 (0.5)	1 (0.8)	0.6963**	3 (1.2)	2 (1.6)	1 (0.8)	0.5614**
**History of Bariatric Surgery, n (%)**	1 (0.1)	0 (0.0)	1 (0.8)	0.0303**	1 (0.4)	0 (0.0)	1 (0.8)	0.3164**
**Concomitant Aspirin use, n (%)**	257 (36.0)	218 (37.0)	39 (31.5)	0.2412^^	82 (33.1)	43 (34.7)	39 (31.5)	0.5893^^
**Concomitant Clopidogrel use, n (%)**	93 (13.0)	82 (13.9)	11 (8.8)	0.1223^^	29 (11.7)	18 (14.5)	11 (8.8)	0.1598^^
**Concomitant Ticagrelor use, n (%)**	2 (0.3)	2 (0.3)	0 (0.0)	0.5141**	0 (0)	0 (0)	0 (0)	NA
**Concomitant use of GI prophylaxis (i.e., PPI or H2A), n (%)**	602 (83.4)	497 (83.5)	105 (82.7)	0.81	214 (84.3)	108 (85.0)	106 (83.4)	0.7509^^
**Comorbidity**								
**Aortic Stenosis (AS), n (%)**	32 (4.4)	27 (4.5)	5 (3.9)	0.7652^^	10 (3.9)	5 (3.9)	5 (3.9)	> 0.9999^^
**Mitral Stenosis (MS), n (%)**	28 (3.9)	23 (3.9)	5 (3.9)	0.9698**	10 (3.9)	5 (3.9)	5 (3.9)	> 0.9999^^
**Chronic obstructive pulmonary disease (COPD**)**, n (%)**	42 (5.8)	30 (5.0)	12 (9.4)	0.0541^^	22 (8.66)	10 (7.87)	12 (9.45)	0.6555^^
**Heart Failure, n (%)**	315 (43.6)	253 (42.5)	62 (48.8)	0.1939^^	111 (43.7)	49 (38.6)	62 (48.8)	0.1001^^
**Hypertension, n (%)**	606 (83.9)	490 (82.4)	116 (91.3)	0.0123^^	228 (89.7)	112 (88.2)	116 (91.3)	0.4077^^
**Diabetes mellitus, n (%)**	490 (67.9)	396 (66.6)	94 (74.0)	0.1021^^	179 (70.5)	85 (66.9)	94 (74.0)	0.2157^^
**Dyslipidemia, n (%)**	395 (54.7)	324 (54.5)	71 (55.9)	0.7654^^	147 (57.8)	76 (59.8)	71 (55.9)	0.5252^^
**Hypothyroidism, n (%)**	120 (16.6)	101 (17.0)	19 (15.0)	0.5799^^	44 (17.3)	25 (19.7)	19 (14.9)	0.3198^^
**Hyperthyroidism, n (%)**	9 (1.2)	8 (1.3)	1 (0.8)	0.6074**	3 (1.18)	2 (1.57)	1 (0.79)	0.5614
**Ischemic heart disease (IHD), n (%)**	190 (26.3)	165 (27.7)	25 (19.7)	0.0616^^	54 (21.3)	29 (22.8)	25 (19.7)	0.5396^^
**Chronic kidney disease (CKD), n (%)**	85 (11.8)	72 (12.1)	13 (10.2)	0.5539^^	27 (10.6)	14 (11.0)	13 (10.2)	0.8387^^
**Cancer, n (%)**	39 (5.4)	33 (5.5)	6 (4.7)	0.7099^^	13 (5.1)	7 (5.5)	6 (4.7)	0.7758^^
**Venous Thromboembolism, n (%)**	45 (6.2)	39 (6.6)	6 (4.7)	0.4386^^	14 (5.5)	8 (6.3)	6 (4.7)	0.5824^^
**Vascular Disease, n (%)**	9 (1.2)	6 (1.0)	3 (2.4)	0.2119**	4 (1.6)	1 (0.8)	3 (2.4)	0.3135**
**Liver disease (any type), n (%)**	34 (4.7)	29 (4.9)	5 (3.9)	0.6509^^	9 (3.5)	4 (3.1)	5 (3.9)	0.7343**
**Stroke, n (%)**	136 (18.8)	121 (20.3)	15 (11.8)	0.0257^^	41 (16.1)	26 (20.5)	15 (11.8)	0.0607^^

*T Test / ^ Wilcoxon rank sum test is used to calculate the *P*-value

^^ Chi square/ ** Fisher’s Exact teat is used to calculate *P*-value

### Outcomes

#### Thrombosis

Thrombotic events occurred in three patients (2.4%) with BMI ≥ 40 compared to five patients (4.0%) in the control group (*p* = 0.46) within 12 months of Apixaban initiation. Logistic regression analysis showed lower odds of thrombotic events in patients with BMI ≥ 40, but that did not reach the statistical significance (OR (95%CI) 0.58 (0.13, 2.51), *p* = 0.46). Moreover, the prevalence of Venous Thromboembolism (VTE) and ischemic stroke were similar between the two groups (OR (95%CI) 0.49 (0.04, 5.51), *p* = 0.56 and OR (95%CI) 0.66 (0.10, 4.02), *p* = 0.64 respectively) (Table 2).

**Table 2:** Regression analysis for the thrombosis after PS matching

Outcomes	Follow-up	Number of outcomes/Total number of patients		*P*-value	Odds Ratio (OR) (95%CI)	*P*-value ^$^
		BMI < 40	BMI ≥ 40			
**Arterial/ Venous Thrombosis**						
**All thrombosis events, n (%) ∆**	**Within 12 months**	5/125 (4.0)	3/126 (2.4)	0.46**	0.58 (0.13,2.51)	0.46
	**> 12 months**	12/123 (9.8)	6/125 (4.8)	0.13^^	0.46 (0.16,1.28)	0.13
**Venous Thromboembolism (VTE), n(%)∆**	**Within 12 months**	2/125 (1.6)	1/126 (0.8)	0.55**	0.49 (0.04,5.51)	0.56
	**> 12 months**	6/124 (4.8)	1/126 (0.8)	0.05**	0.15 (0.01,1.27)	0.08
**Ischemic Stroke, n (%) ∆**	**Within 12 months**	3/125 (2.4)	2/126 (1.6)	0.64**	0.66 (0.10,4.02)	0.64
	**> 12 months**	7/125 (5.6)	5/126 (4.0)	0.54^^	0.70 (0.21,2.26)	0.54

∆ Denominator of the percentage is the total number of patients

**^^** Chi-square/** Fisher test is used to calculate the P-value

$ Propensity score matched used based patient’s age, gender and HAS-BLED score

As well, all thrombosis and ischemic stroke events were similar when patients were followed beyond 12 months (OR (95%CI) 0.46 (0.16, 1.28), *p* = 0.13 and OR (95%CI) 0.70 (0.21, 2.26), *p* = 0.54 respectively). On the other hand, VTE events were higher in patients with BMI < 40; although, it was not statistically significant (OR (95%CI) 0.15 (0.01, 1.27), *p* = 0.08) (Table 2). Of interest, there were no thrombosis events in the sub-group analysis for patients with BMI ≥50 who received Apixaban compared to the control group (Table 3).

**Table 3:** Regression analysis of the sub-group analysis (BMI ≥ 50) after PS matching

Outcomes	Number of outcomes/Total number of patients		*P*-value^$^
	BMI < 40 (***n*** = 24)	BMI ≥ 50 (***n*** = 24)	
**Arterial/ Venous Thrombosis**			
All thrombosis cases, n (%) ∆	0 (0)	0 (0)	NA
Venous Thromboembolism (VTE), n(%)∆	0 (0)	0 (0)	NA
Ischemic Stroke, n (%) ∆	0 (0)	0 (0)	NA
**Bleeding**			
Major Bleeding, n(%)∆	0 (0.0)	1 (4.2)	0.31**
Minor Bleeding, n(%)∆	3 (12.5)	1 (4.2)	0.29**

∆ Denominator of the percentage is the total number of patients

** Fisher test is used to calculate the P-value

$ Propensity score matched used based patient’s age, gender and HAS-BLED score

#### Bleeding

In crude analysis, major bleeding occurred in two patients (1.6%) who had BMI ≥40, compared to five patients (4.0%) in the control group (*p* = 0.24). In the multivariable regression analysis, major bleeding was similar between the two groups (OR (95%CI) 0.39 (0.07, 2.03), *p* = 0.26). In addition, patients who have BMI ≥40 have a similar prevalence of minor bleeding in comparison to the control group (OR (95%CI) 1.27 (0.56, 2.84), *p* = 0.55) as shown in Table 4. The concomitant use of Antiplatelets (i.e., Aspirin, Clopidogrel, and Ticagrelor) and GI prophylaxis were assessed, and both were not significantly different before and after PS matching between the two groups (Table 1).

**Table 4:** Regression analysis for bleeding after PS matching

Outcomes	Number of outcomes/Total number of patients		*P*-value	Odds Ratio (OR) (95%CI)	*P*-value ^$^
	BMI < 40	BMI ≥ 40			
**Bleeding**					
**Major Bleeding, n(%)∆**	5 (4.0)	2 (1.6)	0.24**	0.39 (0.07,2.03)	0.26
**Minor Bleeding, n(%)∆**	12 (9.6)	15 (11.9)	0.55^^	1.27 (0.56,2.84)	0.55

∆ Denominator of the percentage is the total number of patients

**^^** Chi-square/** Fisher test is used to calculate the P-value

$ Propensity score matched used based patient’s age, gender and HAS-BLED score

## Discussion

This exploratory study demonstrated no significant difference in apixaban effectivness between morbidly obese (BMI ≥ 40) patients compared with non-morbidly obese patients with non-valvular AF. In addition, no significant difference in the apixaban safety outcomes between the two treatment groups (BMI < 40 vs. BMI ≥ 40) after apixaban initiation.

The prevalence of thrombotic events in all patients prescribed apixaban was similar between the morbidly obese patients (BMI ≥ 40) and the control group (BMI < 40). In this exploratory study, stroke occurrence was numerically higher among the non-morbid obese patients but not statistically significant. Even though the BMI was significantly different between the two treatment groups, similar rates of thrombotic events between the two treatment groups may be explained by the similar baseline CHA2DS2-Vasc score initially. Consistently, a single-center retrospective study of obese patients (weight > 120 kg vs. < 120 kg) with AF showed a lower incidence of stroke, VTE, and PE in patients using dabigatran, rivaroxaban, or apixaban (2.5% in patients weighing ≥120 kg versus 3.1% in patients weighing < 120 kg (*P* = 0.632)) [23]. Moreover, a retrospective cohort study assessing apixaban effectiveness in morbidly obese (BMI > 40 kg/m^2^ or weight > 120 kg) patients with AF showed no difference between the risk of stroke or Systemic Embolism (SE) between apixaban and warfarin (HR: 0.66 (95% CI 0.32–1.33) [14] These findings could be related to the obesity paradox phenomenon, which refers to the fact that obesity in somepatients with multiple disease comorbidities could be a protective factor leading to a reduction in mortality [24, 25]. The phenomenon is not fully understood, and the evidence available to support this phenomenon’s existence has several limitations [24, 25]. In compared to non-obese patients with known CAD who undergo PCI, Gruberg et al. found that obese patients with known CAD who undergo PCI have a superior outcome in terms of in-hospital complications, cardiac death, and one-year mortality [26]. In addition, several studies reported better outcomes and mortality reduction among obese patients with hypercholesterolemia, hypertension, heart failure, CAD, and chronic obstructive pulmonary disease(COPD) compared to non-obese patients with similar comorbidities [27–29]. Additional research may be required to completely explain these events and improve our understanding of obesity as a cardiovascular disease risk factor. 

Since apixaban does not require routine laboratory measurement of drug level [30], there is a greater concern about under-dosing apixaban in patients who are morbidly obese [31]. But this issue has been refuted by the findings of a previous study reporting that extremes of body weight demonstrated a neglectable effect on the pharmacokinetic and dynamic of apixaban [32]. Therefore, they suggested that apixaban do not require dose adjustment based on weight only [32]. These findings could explain the similar rate of thrombotic events witnessed in our study between morbidly obese patients and non-morbid obese patients using the same dose of apixaban.

In this exploratory study, we observed no significant difference in the incidence of bleeding between morbidly obese patients and non-morbidly obese after controlling for patients; age and HAS-BLED score at the time of apixaban initiation. Furthermore, the major bleeding rate was numerically lower while the minor bleed was numerically higher among the morbidly obese patient group. These similar rates in bleeding among the treatment groups may be attributed to the comparable rates of concomitant antiplatelet use, HAS-BLED score, and renal or liver function at baseline. A single centered retrospective cohort study included patients with a BMI ≥ 40 kg/m^2^ who were prescribed apixaban, rivaroxaban, or warfarin for either VTE or AF showed lower rates of major bleeding among patients using apixaban and rivaroxaban than warfarin [13]. Similar to our finding, two retrospective studies compared DOAC safety in patients with AF and BMI ≥ 40 kg/m^2^; both studies showed non-significantly lower rates of major bleeding with DOACs [12, 15]. In a prior study, patients taking DOACs had a higher rate of minor bleeding than those taking warfarin [12].

It is noteworthy that most of the previous studies compared either multiple DOAC agents or apixaban to warfarin [12–14], while in our study, both groups were on apixaban but stratified based on BMI. As far as we know, this is one of the first studies that compared the use of apixaban among morbid obese patients to non-morbid obese using BMI ≥40 kg/m^2^. This study also minimized the effect of the confounders using propensity score matching between the two groups. However, our study remains to have some limitations. First, it is a single-centered retrospective study that may introduce some bias, and residual confounders may still exist and cannot be fully excluded. Second, given the nature of the study, we could not assess the patients’ adherence to apixaban or concomitant use of medication and interactions that may influence the result during the follow-up period. All these limitations may limit the generalizability of the study results.

## Conclusion

This study showed similar effectiveness and safety of apixaban use in both morbid obese and non-morbid obese patients with non-valvular AF. These findings suggest that apixaban might be an effective and safe option to be used for non-valvular AF stroke prevention in morbidly obese patients. This exploratory study adds to the body of evidence available suggesting the efficacy and safety of apixaban use for AF in patients with extreme weights. However, a larger randomized controlled trial with a longer follow-up period needs to confirm our findings.

## Data Availability

The datasets used and/or analyzed during the current study are available from the corresponding author on reasonable request.

## Abbreviations

AF: Atrial fibrillation
BMI: Body mass index
CI: Confidence intervals
DVT: Deep vein thrombosis
OR: Odds ratio
DOAC: Direct oral anticoagulation
SE: Systemic embolism
HR: Hazard ratio
PS: Propensity score
PRBC: Packed red blood cells
Q1: 1st quartile
Q3: 3rd quartile
SD: Standard deviation
ISTH: International Society on Thrombosis and Hemostasis
PE: Pulmonary embolism
GI: Gastrointestinal
KAMC: King Abdulaziz Medical City
ICD: International Classification of Disease
aPTT: Partial thromboplastin time
eGFR: Estimated glomerular filtration rate
